# The complete mitochondrial genome of an enigmatic predaceous springtail *Metisotoma macnamarai* from northeast China

**DOI:** 10.1080/23802359.2019.1704660

**Published:** 2020-01-10

**Authors:** Zhijng Xie, Haifeng Yao, Mikhail Potapov, Jie Dong, Donghui Wu, Stefan Scheu, Xin Sun

**Affiliations:** a Key Laboratory of Wetland Ecology and Environment, Northeast Institute of Geography and Agroecology, Chinese Academy of Sciences, Changchun, China;; b University of Chinese Academy of Sciences, Beijing, China;; c J.F. Blumenbach Institute of Zoology and Anthropology, Animal Ecology, University of Göttingen, Göttingen, Germany;; d Senckenberg Museum of Natural History Görlitz, Görlitz, Germany;; e Moscow State Pedagogical University, Moscow, Russia;; f College of Plant Protection, Nanjing Agricultural University, Nanjing, China

**Keywords:** mt genome, Collembola, Isotomidae, phylogeny, Holarctic

## Abstract

The complete mitogenome of *Metisotoma macnamarai* (Folsom 1918) (Collembola, Isotomidae), a member of obligatory predaceous genus *Metisotoma* Maynard, 1951, was sequenced. It has a length of 15,177 bp, comprising 13 protein-coding genes, 22 tRNAs, and 2 rRNAs. The mitogenome has the following base composition: A = 37.1%, T = 33.3%, G = 11.8%, and C = 17.4%. Phylogenetic analysis using maximum likelihood (ML) indicates that *M. macnamarai* clusters as sister taxon to the genus *Isotomurus*, with high statistical support.

Soil animals are important drivers of decomposition processes and forming soil microstructure in terrestrial ecosystems (Petersen 2002; Potapov et al. 2016). Collembola (springtails) are numerically dominant microarthropods with more than 9000 species reported in the world (Bellinger et al. 1996–2019). With more than 1439 species reported (Bellinger et al. 1996–2019), the family Isotomidae is among the most diverse Collembola family occurring in virtually any habitat of the Palearctic region (Potapov 2001). The genus *Metisotoma* (Maynard 1951) is the only veritable predator among Collembola with associated aggressive behavior (Macnamara 1924; Potapov et al. 2018). Its predation is reflected in a remarkably large head, short and thin antennae, and peculiar mouth parts (Cassagnau 1972). *Metisotoma macnamarai* (Folsom 1918) is a typical representative of the genus and so far was recorded in Canada and Russian Far East. In the course of our study of Collembola of Changbai Mountains (northeast China), we collected this species and sequenced its mitogenome. Mitogenomic studies perform well in constructing phylogenetic relationships of Collembola (Carapelli et al. 2014, 2019; Leo et al. 2019). However, it has been little used at the intra-family level because of limited available mitogenomic data in public datasets. Here, we display the complete mitogenome of *M. macnamarai* and explore its phylogenetic placement among Isotomidae.

DNA was extracted from one individual collected from northeast China (Changbai Mountains, 42.058°N, 128.066°E, 1850 m alt., 13 Sep. 2015, 10 cm deep coniferous forest soil, leg. Donghui Wu), and its DNA was deposited in the Nanjing Agricultural University, Nanjing, China (NCBI BioSample accession SAMN13483728; Voucher No. C4). Nondestructive DNA extraction was performed using Ezup Column Animal Genomic DNA Purification Kit (Sangon Biotech, Shanghai, China) following the manufacturer’s standard protocols. Primer pairs LCO1490 and HCO2198 (Folmer et al. 1994) were used to amplify DNA sequences of cytochrome c oxidase subunit I (COXI) following Zhang et al. (2014). PCR products were visualized on a 1% agarose gel. Successful products were purified and sequenced in both directions by Majorbio (Shanghai, China) on the ABI 3730XL DNA Analyzer (Applied Biosystems). Sequences were assembled using Sequencher 4.5 (Gene Codes Corporation, Ann Arbor, USA), preliminarily aligned using MEGA 7.0 (Kumar et al. 2016), and thereby generated a COXI alignment with 658 bp in length as seed sequence in the assembly (accession number: MN787750).

The DNA concentration was measured by Qubit 3.0 using Q33230 Qubit™ 1X dsDNA HS Assay Kit. The species with other seven species generated a pool, with equal DNA concentration per species. Each library was sequenced with an insert size of 350 bp on HiSeq X Ten platform (Tianjin Novogene Bioinformatics Technology Co., Ltd, China) generating 150 bp paired-end reads. Non-mitochondrial reads were filtered using NextGenMap 0.5.5 (Sedlazeck et al. 2013) and SAMtools 0.1.18 (Li et al. 2009), and removed from raw data. Assemblies were performed on NOVOPlasty v2.7.0 (Dierckxsens et al. 2017) using COXI sequence. The chimera detected with VSEARCH (Rognes et al. 2016; Zhang et al. 2019). Annotations were performed using the MITOS web server (Bernt et al. 2013) and tRNAs gene limits were rechecked with tRNA scan-SE (Lowe and Eddy 1997; Bernt et al. 2013). Sequences were deposited in GenBank (accession number: MN592792).

Amino-acid sequences of each protein-coding gene (PCG) in mitochondria were aligned using MAFFT v.7.394 (Nakamura et al. 2018), and automated alignment trimming (-automated1) was performed in trimAL v.1.4 (Capella-Gutiérrez et al. 2009). Final concatenated supermatrices were performed in FASconCAT-G v1.04 to get the amino acid sequences of 13 PCGs (Kück and Longo 2014). To infer phylogeny, all partition and substitution models on supermatrices 13 PCGs were determined by ModelFinder (Kalyaanamoorthy et al. 2017) and built-in IQ-TREE v1.6.3 (Nguyen et al. 2015) with 1000 ultrafast bootstraps (UFBoot; Hoang et al. 2018).

The total length of the circularized mitochondrial genome is 15,177 bp. Typical mitogenomic features comprise 13 PCGs, 22 tRNAs, and two rRNAs. A non-coding A + T-rich region was detected, involved in the regulation of replication and transcription processes (166 bp long) (Goddard and Wolstenholme 1978; Carapelli et al. 2008). The base composition is 37.1% adenine, 33.3% thymine, 11.8% guanine and 17.4% cytosine.

In the phylogenetic tree of concatenated amino-acid sequences from 13 PCGs, nodes were well-supported by high bootstrap values (Figure 1). In the widely accepted classification of Isotomidae (Potapov 2001), three genera *Metisotoma*, *Isotomurus* and *Folsomotoma* belong to the subfamily Isotominae, while others belong to Anurophorinae among the selected genera in our study. However, in the phylogenetic tree only two species, *M*. *macnamarai* and *Isotomurus maculatus* (Schäffer 1896), clustered together, without the species *Folsomotoma octooculata* (Willem 1901). Middle-sized furca with rather slender crenulated dens indicated the subfamily Isotominae, but reduced s-chaetotaxy of the body and the intermediate number of setae on manubrium suggests Anurophorinae. The evolutionary relationships of these two subfamilies need to be explored in more detail as soon as more mtDNA sequences are being available.

**Figure 1.**
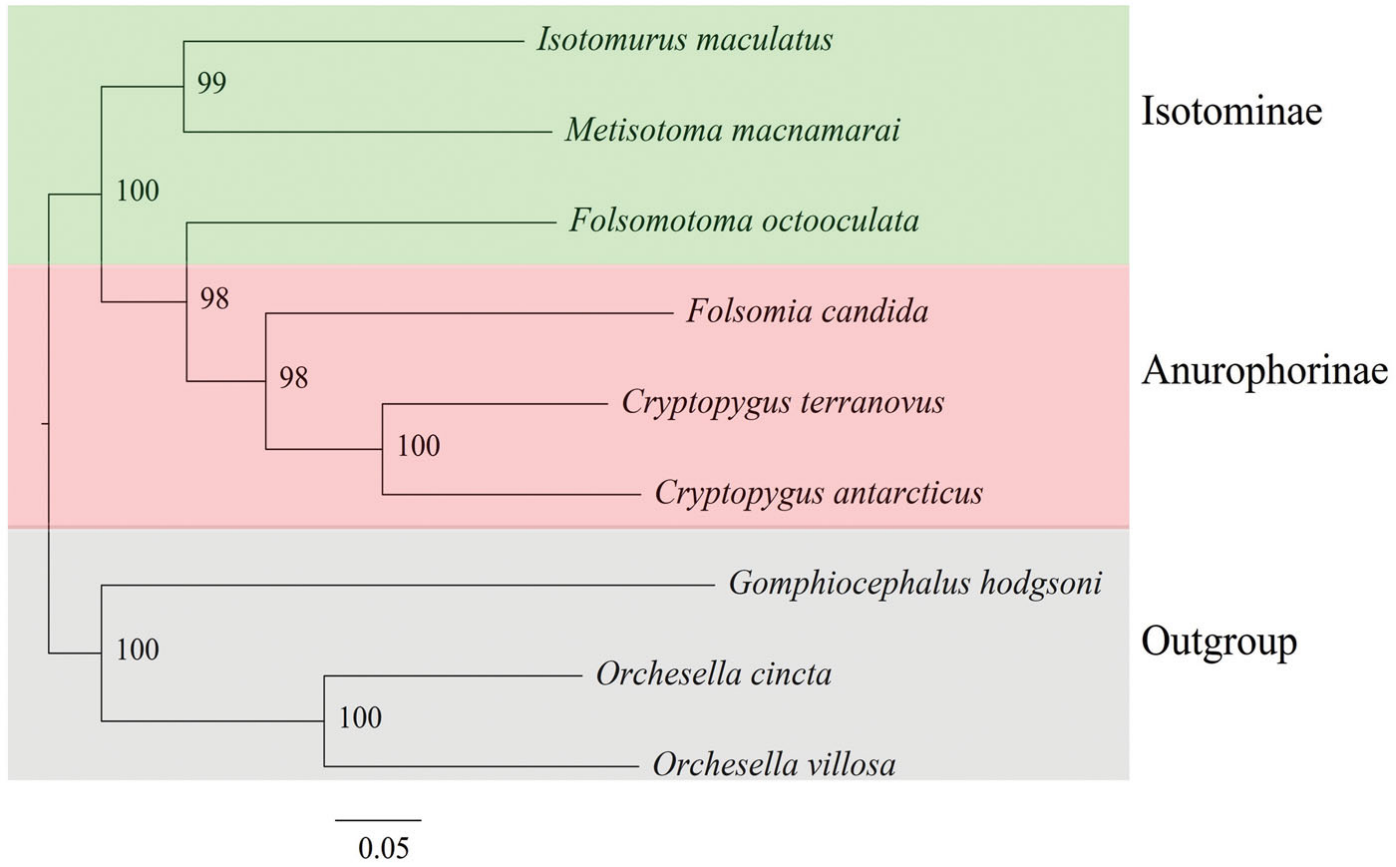
Maximum likelihood phylogenetic tree inferred from partitioned amino acid sequences of 13 PCGs. Bootstrap support values are shown in the nodes. The following species were used for the phylogenetic analysis: *Metisotoma macnamarai* MN592792 (Isotomidae), *Isotomurus maculatus* MK509021 (Isotomidae), *Cryptopygus antarcticus* NC_010533 (Isotomidae), *Cryptopygus terranovus* KX863671 (Collembola, Isotomidae), *Folsomia candida* KU198392 (Isotomidae), *Folsomotoma octooculata* NC_024155 (Isotomidae), *Orchesella cincta* NC_032283 (Entomobryidae), *Orchesella villosa* EU016195 (Entomobryidae), *Gomphiocephalus hodgsoni* AY191995 (Hypogastruridae).
